# From Sphygmomanometer to Lungs: Unveiling Mercury Poisoning in Emergency Department

**DOI:** 10.7759/cureus.65699

**Published:** 2024-07-29

**Authors:** Preethy Koshy, Rajshree Devi Seram, Aditya Pundkar

**Affiliations:** 1 Emergency Medicine, Datta Meghe Institute of Higher Education and Research, Jawaharlal Nehru Medical College, Wardha, IND; 2 Orthopedics, Datta Meghe Institute of Higher Education and Research, Jawaharlal Nehru Medical College, Wardha, IND

**Keywords:** toxic epidermal necrolysis (ten), acute respiratory distress syndrome, chemical pneumonitis, heavy metal poisoning, chelating agents, mercury poisoning

## Abstract

Mercury, a ubiquitous heavy metal, poses a significant threat to human health. Intravenous mercury poisoning is an uncommon but critical medical emergency. The nature and severity of its toxic effects depend on the form of mercury encountered: elemental, inorganic, or organic. It can affect almost all organ systems in the body. Chelating agents are the primary treatment for symptomatic mercury poisoning. This case report is about a 27-year-old male patient who presented to the emergency department with an alleged history of intravenous injection of mercury as an attempt at suicide, followed by breathlessness, chest pain, vomiting, and high-grade fever. He was managed with chelating therapy, non-invasive ventilation, and other supportive measures and was discharged home. After five days of discharge, he presented with fever and rashes and was diagnosed with toxic epidermal necrolysis (TEN). In spite of all aggressive management, he succumbed to death after four days of re-admission. Early intervention can significantly improve the chances of recovery. However, even with successful treatment, some individuals may experience long-term complications.

## Introduction

Mercury is a heavy metal known to cause toxicity and has been linked to public health issues. The chemical structure of mercury determines its biological behavior, pharmacokinetics, and clinical importance. The toxicity produced in humans differs depending on the form of mercury, the rate, and the dose of exposure. Three forms of mercury are found in the environment: organic mercury (ethyl mercury and methyl mercury), inorganic mercury (mercuric mercury), and elemental mercury (poisonous as vapor). All of these forms have detrimental effects on one’s health [1]. The brain is predominantly the target organ for mercury. In vivo, there is some interconversion of the several forms of mercury. Mercury disrupts normal cell function by attaching to internal sulfhydryl-containing proteins and enzymes [2].

Elemental mercury is found in dental amalgams, fluorescent light tubes, sphygmomanometers, thermometers, and mercury added to latex paint. It is extremely volatile, lipophilic, and rapidly oxidized to other forms. It is quickly absorbed through mucous membranes and the lungs. Patients who breathe in mercury vapor may develop acute respiratory distress and chemical pneumonitis. Long-term exposure may harm the kidneys and central nervous system [3].

Metallic mercury poisoning, administered intravenously for suicide, is quite rare. When metallic mercury is injected, the amount of mercury in the blood can rise quickly and eventually spread throughout the body. As the poisoning progresses, the clinical signs change and might involve the kidney, liver, and gastrointestinal tract in addition to respiratory symptoms [4]. Mercury toxicity can cause neurological symptoms such as tremor, sensory disruption, and cognitive impairment [5].

Skin manifestations, in the form of allergic contact dermatitis (ACD), of mercury toxicity are well known with oral, direct dermal and inhalational route. It is characterized by pruritic and painful eczematous eruptions mostly seen in skin folds and then spreads to whole of the body. Toxic epidermal necrolysis (TEN) is a rare mucocutaneous disease caused by medications like non-steroidal anti-inflammatory drugs, several antibiotics, anticonvulsants, etc. It is rarely seen as a complication of mercury poisoning or as a side effect of its chelating agents. It is characterized by lesions involving mucous membrane and skin which progresses to blisters and then epidermal detachment [6]. We hereby present a rare case of suicidal intravenous mercury injection by a 27-year-old man.

## Case presentation

A 27-year-old male patient was brought to the emergency room (ER) with an alleged history of self-injection of mercury intravenously around 7 ml five days ago, which he had extracted from the sphygmomanometer. This was followed by breathlessness, which was insidious in onset and gradually progressive, making him feel breathless even at rest at the time of presentation. It was associated with a dry cough and chest pain. He also complained of a high-grade intermittent fever of four days duration with multiple episodes of vomiting and vomitus containing food particles. His history revealed multiple suicidal ideations, but he was not on any psychiatric medications.

On examination, his respiratory rate was 32 cycles per minute with an oxygen saturation of 94% in room air. He was tachycardic with a pulse rate of 132 beats per minute and a blood pressure of 120/80 mm of mercury. His random blood sugar was within normal limits. He was febrile, with a temperature of 102-degree Fahrenheit. The respiratory examination showed bilateral coarse crepitations all over the lung field. The rest of the systemic exams were within normal limits. He was managed with non-invasive ventilation, intravenous antibiotics, antipyretics, inhaled corticosteroids, levosalbutamol and N-acetyl cysteine, and other supportive measures.

**Figure 1:**
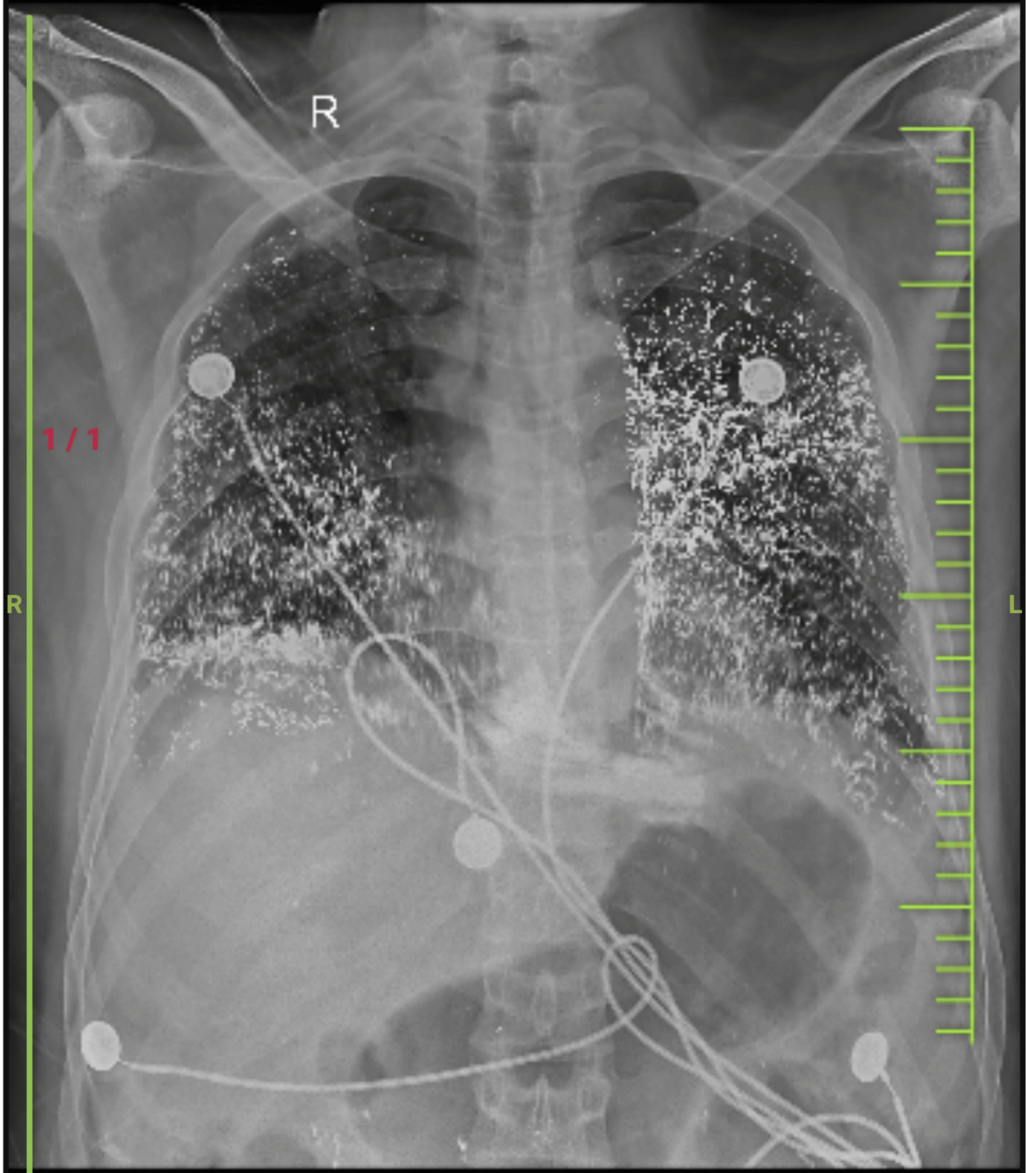
X-ray of patient showing linear radio-opacities in bilateral lung fields

His blood investigations revealed mild hemolytic anemia (hemoglobin: 10.6 g/dl) with neutrophilic leukocytosis (total leukocytic count: 20,400 cells/mm^3^). His serum and urine mercury levels were raised (250 μg/l), and his liver and renal function tests showed mild derangement. An electrocardiogram revealed sinus tachycardia. His chest X-ray showed peripherally enhanced opacities suggestive of acute respiratory distress syndrome with mercury artifacts, as shown in Figure 1.

**Figure 2:**
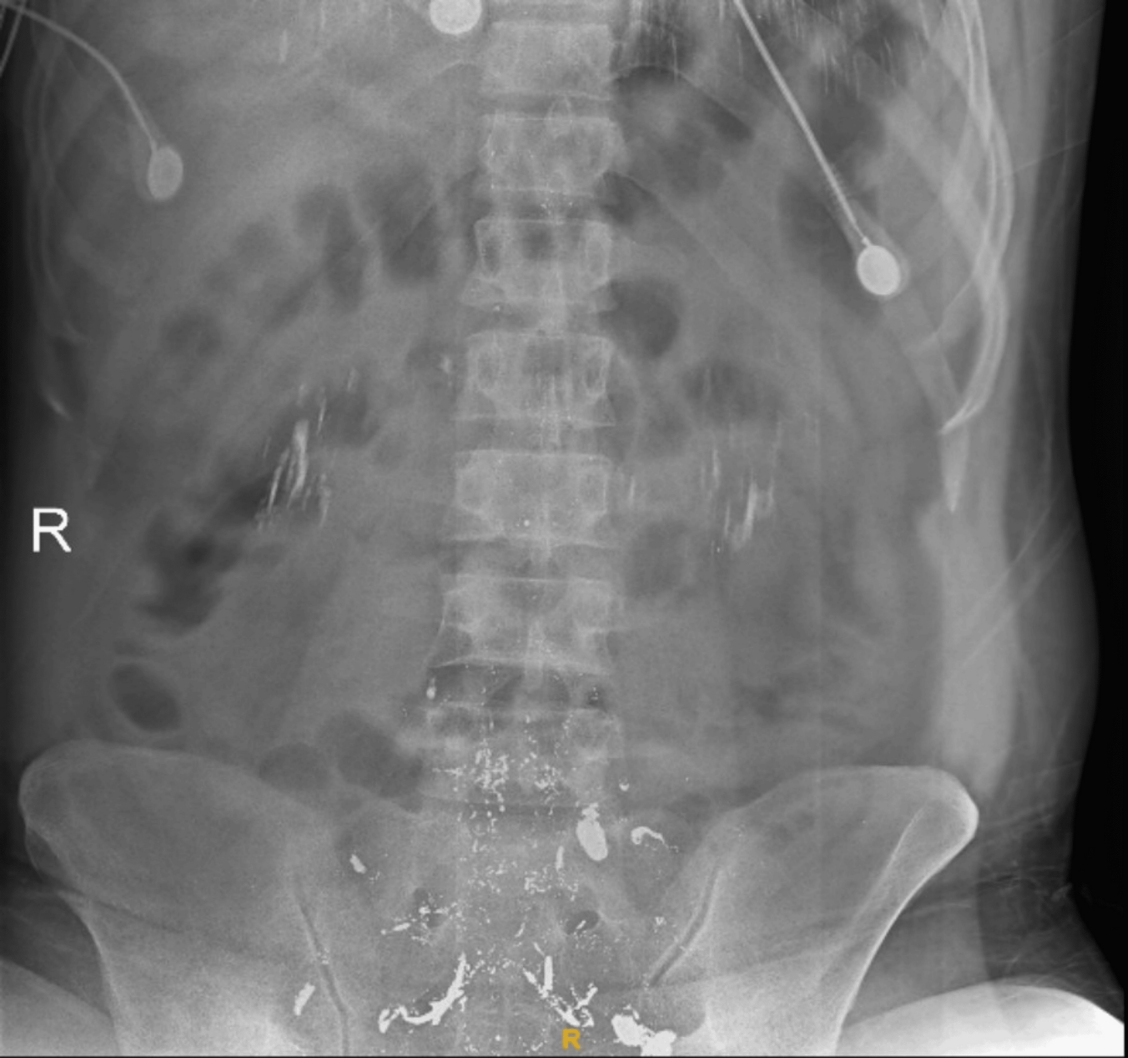
X-ray erect abdomen showing multiple variable sized radio-opacities in bilateral renal fossae and pelvis

X-ray abdomen erect done showed multiple variable sized radio-opacities with metallic density in the region of bilateral renal fossae and pelvis as shown in Figure 2. 

Thus, a diagnosis of mercury toxicity with acute respiratory distress syndrome with sepsis was made. He was admitted to an intensive care unit. The fever profile, comprising dengue, Widal, scrub typhus, and leptospirosis, was found to be negative. His blood culture revealed the growth of a coagulase-negative staphylococcus. He was managed with dimercaprol 150 mg intramuscularly twice daily, intravenous antibiotics, antipyretics, non-invasive ventilation, and other supportive measures. Multiple blood transfusions were done in view of a drop in hemoglobin. His two-dimensional echo showed normal biventricular function with a normal ejection fraction. Over the course of his hospital stay, he became vitally and hemodynamically stable and was discharged home.

**Figure 3:**
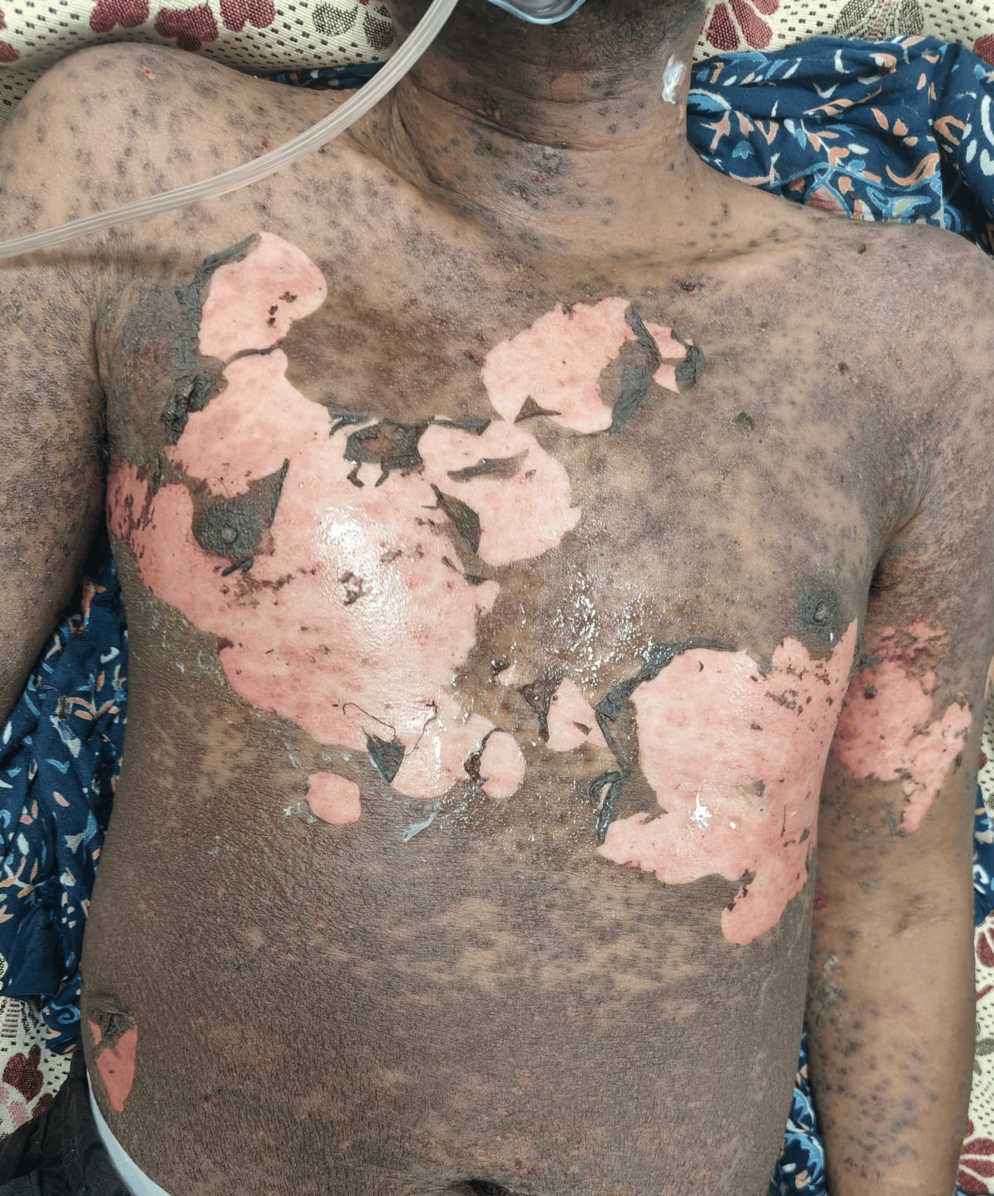
Figure showing maculo-papular rashes with desquamation

However, after five days of discharge, he again presented to the ER with complaints of fever and rashes. Rashes were present initially in the palms and soles and then extended all over the body, involving the oral cavity and genitals with vesicles and desquamation of skin, as shown in Figure 3.

He was toxic-looking with tachypnea, tachycardia, and a blood pressure of 90/60 mm Hg. He was immediately started on intravenous crystalloids, antibiotics, and other supportive measures. A dermatology consultation was given suspecting TEN, and they had advised an immunosuppressant (cyclosporine). He was again admitted to the intensive care unit. A skin biopsy done confirmed the diagnosis of TEN. He was planned for plasmapheresis and intravenous immunoglobin. However, due to his deteriorating condition, his relatives were not willing for any further intervention, and he succumbed to death after four days of admission.

## Discussion

Mercury compounds exert toxic health effects by different mechanisms, such as disruption of microtubule formation, inhibition of enzymes, changing intracellular calcium balance and membrane potential, altering cell membrane integrity, inducing oxidative stress, inhibition of protein synthesis, and disrupting immune functions. Different forms of mercury compounds have different presentations and adverse effects [7].

When elemental mercury vapors are inhaled, it can result in both acute symptoms like fever, chills, cough, and breathlessness, and gastrointestinal issues like nausea, vomiting, diarrhea, and dysphagia, along with a metallic taste. The clinical signs of intoxication include dry cough, hypoxemia, dyspnea, and chest discomfort [8], as seen in our patient. Higher amounts of mercury vapor can result in pneumonitis, bronchiolitis, and necrotizing bronchitis. Additionally, it may develop into respiratory failure, pulmonary edema, and even death. Subcutaneous emphysema, pneumomediastinum, and numerous pneumothoraces are among the complications [9]. Severe pulmonary complications, such as persistent restrictive pulmonary illnesses and interstitial fibrosis, can emerge in survivors [8].

Systemic microembolism and acute pulmonary embolism with respiratory failure are caused by the intravenous injection of mercury [9]. Arteriolar embolism can occur as a result of the stable nature of metallic mercury in the blood, which can persist in the form of mercury globules for an extended period of time [10]. Following injection, a significant amount of mercury travels through the pulmonary capillary bed, gains entry into the pulmonary circulation via the affected vein, and eventually spreads throughout the body [4].

Hemolysis caused by mercury exposure has been demonstrated to increase the incidence of anemia [11]. Mercury triggers a complex chain reaction that damages and eventually kills cells. It enters red blood cells (RBCs) and causes a reduction in endogenous antioxidant activity and an increase in pro-oxidant activity, which in turn causes peroxidative RBC membrane breakdown and hemolysis [12], as seen in our patient.

There are several methods to effectively remove mercury from the body. In order to stop mercury poisoning from spreading in the early stages, ligation and excision of the impacted arteries can be employed [4]. Metal chelation treatment plays a very important role for patients exposed to mercury. It alleviates clinical symptoms and lowers mercury levels in the blood. The two main chelating drugs used in clinical settings are dimercaptopropanesulfonic acid (DMPS) and dimercaptosuccinic acid (DMSA) [13]. It has been observed that hemodialysis and plasma exchange are efficient treatments for lowering blood mercury levels, along with chelation therapy [14].

Cutaneous manifestations of mercury toxicity are reported in literature so far as ACD [6]. Our patient presented with fever and rashes within 10 days of administration of DMPS. Differential diagnoses we considered were viral exanthema, cutaneous manifestation of mercury toxicity itself, or DMPS-induced TEN. His fever profile was negative, thus eliminating infective etiology. Most common side effects of DMPS reported are hypotension and gastrointestinal. TEN, as a side effect has not been reported yet. However, since DMPS contains a sulfonate group, which is also found in glibenclamide and sulfamethoxazole, this adverse effect may be expected [6]. Thus, the cutaneous manifestation seen in our patient may be attributed to DMPS or due to mercury toxicity itself.

## Conclusions

Elemental mercury poisoning administered intravenously for suicide is uncommon but serious. It can be misdiagnosed because symptoms can be vague, and the cause may not be immediately apparent. Numerous harmful outcomes, such as corrosive damage, serious gastroenteritis, neurological manifestations, acute renal failure, circulatory collapse, acute respiratory distress syndrome, and finally death, can result from its toxicity. So early identification and initiation of treatment are crucial in such cases. Chelation therapy is a common treatment, but other methods like hemodialysis and plasma exchange may also be necessary to eliminate mercury from the body. Chelation with DMPS cannot be considered harmless and should be administered in cases with proven toxicity only. However, complete removal of mercury within a short time period is challenging, and residual mercury can cause chronic problems in various organs. So, it is necessary to conduct long-term follow-ups to identify complications and outcomes related to mercury toxicity.
